# Evaluation of Changes in US Health Insurance Coverage for Individuals With Criminal Legal Involvement in Medicaid Expansion and Nonexpansion States, 2010 to 2017

**DOI:** 10.1001/jamahealthforum.2022.0493

**Published:** 2022-04-08

**Authors:** Benjamin A. Howell, Laura Hawks, Emily A. Wang, Tyler N. A. Winkelman

**Affiliations:** 1 SEICHE Center for Health and Justice, Yale School of Medicine, New Haven, Connecticut; 2 Center for Advancing Population Science, Medical College of Wisconsin, Milwaukee; 3 Health, Homelessness, and Criminal Justice Laboratory, Hennepin Healthcare Research Institute, Minneapolis, Minnesota

## Abstract

This cross-sectional study compares changes in health insurance coverage from 2010 to 2017 for low-income US adults with criminal legal involvement in states that did and did not adopt the Medicaid expansion provision of the Affordable Care Act.

## Introduction

Before the Affordable Care Act (ACA) was implemented, more than 80% of US individuals with criminal legal involvement were uninsured. Low health insurance coverage contributes to poor health care access among a population with high rates of physical and behavioral health conditions.^1^ Although health insurance coverage increased nationally for people with criminal legal involvement after the ACA was enacted,^2^ the direct effect of Medicaid expansion has not yet been quantified. In this study, we compared changes in insurance coverage for low-income adults with criminal legal involvement in states that did and did not adopt the ACA Medicaid expansion provision.

## Methods

Because this cross-sectional study used deidentified secondary data from the National Survey on Drug Use and Health (NSDUH), institutional review board approval was not required per Yale University policy. Survey participants provided informed consent for the NSDUH interview. Detailed methods are provided in the eMethods in the Supplement. This study followed the STROBE reporting guideline.

We used restricted data for 2010 to 2017 from NSDUH, a cross-sectional, nationally representative survey of noninstitutionalized individuals aged 12 years or older. We limited our sample to adults aged 18 to 64 years who reported (1) a household income of 138% of the federal poverty level or less and (2) past-year criminal legal involvement (being arrested and booked, paroled, or on probation).

For our exposure of interest, we generated a variable that captured whether an individual resided in a state during a quarter-year in which Medicaid expansion was available (eTable in the Supplement). For our primary outcome of interest, individuals were categorized as insured if they reported being enrolled in private, Medicaid, or other health insurance (including Medicare, Tricare, or the Veterans Health Administration).

Using a difference-in-differences (DiD) methodology, we estimated changes in insurance coverage associated with Medicaid expansion for low-income adults with criminal legal involvement. We used a multivariable linear probability model comparing changes in insurance coverage before and after policy implementation between Medicaid expansion and nonexpansion states. The adjusted model controlled for state and quarter-year fixed effects, age, sex, race and ethnicity, marital status, and employment status. We clustered SEs at the state level. We also reported changes in Medicaid, private, and other insurance coverage before and after ACA implementation for expansion and nonexpansion states. The parallel trend assumption was confirmed by visual inspection and by statistical testing of trends in the pre-ACA period for insurance coverage rates between expansion and nonexpansion states.

Analyses occurred from March 2020 through November 2021 using Stata/SE version 15 (StataCorp) and were completed in a Federal Statistical Research Data Center run by the US Census.

## Results

Our sample comprised 9910 individuals; 6617 (62%) were men, and the mean (SD) age was 34 (9.8) years. The proportion of insured low-income adults with criminal legal involvement increased in both expansion and nonexpansion states after ACA implementation (Figure). In our adjusted DiD analysis, Medicaid expansion was associated with a 14.9–percentage point increase (95% CI, 5.4 to 24.3; *P* = .003; Table) in insurance coverage. This difference was primarily attributable to a larger increase in Medicaid coverage in expansion vs nonexpansion states (DiD, 19.1 percentage points [95% CI, 10.2 to 28.0]; *P* < .001). No significant difference in private insurance was noted with Medicaid expansion (DiD, −1.1 percentage points [95% CI, −5.9 to 3.6]; *P* = .63).

**Figure.**
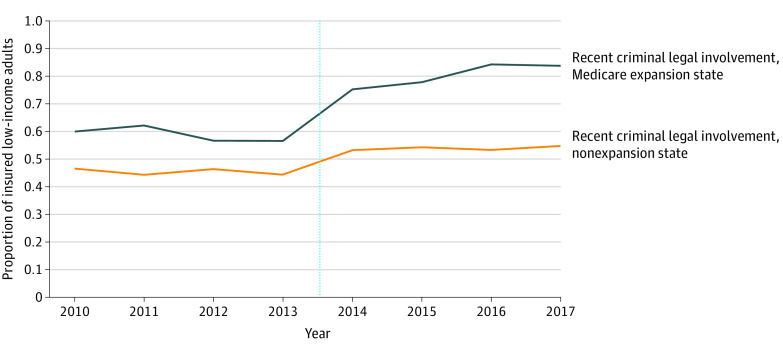
Proportion of Low-Income Adults With Criminal Legal Involvement Who Were Insured in Medicaid Expansion and Nonexpansion States, 2010 to 2017 The vertical dotted line indicates the date of expansion for most expansion states on January 1, 2014.

**Table.** Changes in Insurance Coverage and Insurance Type for Low-Income Adults With Criminal Legal Involvement Before and After Implementation of the ACA in Medicaid Expansion and Nonexpansion States, 2010 to 2017

Insurance	Medicaid expansion states			Nonexpansion states			DiD estimate, percentage points (95% CI)	
	Adults with coverage, % (95% CI)		Difference, percentage points (95% CI)	Adults with coverage, % (95% CI)		Difference, percentage points (95% CI)	Unadjusted	Adjusted
	Pre-ACA	Post-ACA		Pre-ACA	Post-ACA			
Any coverage	59.2 (51.9 to 66.5)	82.5 (80.2 to 82.6)	23.4 (17.2 to 29.5)	45.4 (43.2 to 47.7)	54.2 (48.7 to 59.6)	8.7 (4.1 to 13.4)	14.6 (6.9 to 22.3)	14.9 (5.4 to 24.3)
Medicaid	37.0 (30.4 to 43.5)	61.4 (57.9 to 64.9)	24.5 (16.6 to 32.3)	24.7 (21.7 to 27.9)	29.6 (27.0 to 32.4)	6.2 (2.3 to 10.0)	18.3 (9.6 to 27.0)	19.1 (10.2 to 28.0)
Private	13.3 (11.1 to 15.5)	14.0 (12.0 to 16.0)	0.7 (−1.5 to 2.9)	14.7 (12.6 to 17.3)	17.2 (15.1 to 19.6)	1.5 (−2.3 to 5.4)	−0.8 (−5.2 to 3.6)	−1.1 (−5.9 to 3.6)
Other^a^	5.6 (3.7 to 7.6)	9.7 (6.6 to 12.8)	4.1 (1.5 to 6.7)	3.2 (1.5 to 4.8)	5.9 (2.8 to 9.1)	5.7 (−2.0 to 15)	1.3 (−1.9 to 4.6)	1.5 (−1.5 to 4.5)

Abbreviations: ACA, Affordable Care Act; DiD, difference-in-differences.

^a^ Other includes coverage via Medicare, Tricare, or the Veterans Health Administration.

## Discussion

In this cross-sectional study of NSDUH survey data, Medicaid expansion was associated with a large increase in health insurance coverage for low-income adults with recent criminal legal involvement. According to US Census and other publicly available data, states that have not expanded Medicaid include more than 100 million individuals and have higher incarceration rates than expansion states. Our results suggest that Medicaid expansion in these states could disproportionately benefit people with criminal legal involvement through expanded access to treatment for chronic conditions (eg, behavioral health conditions) and outcomes beyond population health (eg, increased employment and reduced crime).^3,4,5,6^

Study limitations include self-reported outcomes, exclusion of unsheltered homeless or institutionalized individuals from NSDUH, and potential selection bias among survey respondents.

## Notes

Supplement. **eMethods.** Expanded Discussion of Methods **eTable.** State Categorization of Medicaid Expansion by Quarter-Year
