# Supplementary material for: Evaluation of Changes in US Health Insurance Coverage for Individuals With Criminal Legal Involvement in Medicaid Expansion and Nonexpansion States, 2010 to 2017
Source: JAMA Health Forum. 2022 Apr 8;3(4):e220493. doi: 10.1001/jamahealthforum.2022.0493 (PMC8994123; doi:10.1001/jamahealthforum.2022.0493)
Supplement: Supplement. — eMethods. Expanded Discussion of Methods eTable. State Categorization of Medicaid Expansion by Quarter-Year [file jamahealthforum-e220493-s001.pdf]

Supplementary Online Content

Howell BA, Hawks L, Wang EA, Winkelman TNA. Evaluation of changes in US health insurance coverage for individuals with criminal legal involvement in Medicaid expansion and nonexpansion states, 2010 to 2017. *JAMA Health Forum*. 2022;3(4):e220493. doi:10.1001/jamahealthforum.2022.0493

eMethods. Expanded Discussion of Methods

eTable. State Categorization of Medicaid Expansion by Quarter-Year

This supplementary material has been provided by the authors to give readers additional information about their work.

© 2022 Howell BA et al. *JAMA Health Forum*.

eMethods. Expanded Discussion of Methods

We used restricted data from the National Survey on Drug Use and Health (NSDUH) from 2010-2017. Sponsored by the Substance Abuse and Mental Health Services Administration (SAMHSA), the NSDUH is a cross-sectional, nationally representative, annual survey of non-institutionalized residents of the United States age 12 years and older. The NSDUH interviews around 65,000 individuals a year using a combination of telephone, in-person, and computer-assisted survey techniques. Across all years included in this study, NSDUH weighted screening response rates are reported as greater than 75% and weighted interview response rates greater than 67%. For this study we used restricted-use NSDUH data to utilize state-level variables that are not available in the public-use data file. SAMHSA provides access to a restricted-use data file to approved researchers through the US Census' Federal Statistical Research Data Center system.

Study Sample

To target our analysis on the ACA's Medicaid expansion, we limited our sample to adults aged 18-64, with household income ≤138% the federal poverty level. We then further limited our cohort to individuals with past-year criminal legal involvement. We defined presence of past year criminal legal involvement based on responses to questions regarding past year arrest (excluding minor traffic violations) and being on community correctional supervision (parole or probation) in the past year.

Primary exposure of interest: Medicaid Expansion

We defined states that expanded Medicaid between 2010-2017 using data from the Kaiser Family Foundation. To account for differences in when states implemented Medicaid expansion, we defined exposure to Medicaid expansion by state of residence and quarter-year of survey administration. Residence in a state after Medicaid expansion was defined as living in a state for any quarter year after Medicaid expansion was implemented. See supplementary eTable 1 for state-by-state quarter year Medicaid expansion exposure definition used in our analysis.

Primary outcomes of interest: Insurance coverage

We categorized respondents as insured if, at the time of the survey, they were enrolled in a private or public health insurance plan. We subsequently categorized their insurance coverage as being Medicaid coverage, private insurance, or other (e.g.,. Tricare, Veterans Health Administration, or Medicare).

Covariates

We adjusted for respondent age, gender, race and ethnicity, marital status, and employment status in our models. Age was included as a continuous variable. Gender categories were based on self-report, and individuals were categorized as either male or female. Self-reported race and ethnicity categories included non-Hispanic Black, Hispanic, non-Hispanic White, or Other. The Other category includes respondents categorized as non-Hispanic Native American/Alaskan Native, non-Hispanic Native Hawaiian/Pacific Islander, and non-Hispanic Asian, as well as respondents who reported more than one race. Sample size precluded us from further refining "Other" race and ethnicity. We defined employment status as unemployed, employed part-time, employed full-time, or not in labor force.

© 2022 Howell BA et al. *JAMA Health Forum*.

Analysis

We estimated weighted proportions of sociodemographic characteristics in those who resided in Medicaid expansion and non-expansion states. Next, to estimate the impact of Medicaid expansion in individuals residing in a state that expanded Medicaid, we used difference-in-differences (DiD) methods frequently used to analyze the Affordable Care Act and its Medicaid expansion provision. DiD is a quasi-experimental approach to estimate the impact of an intervention by comparing outcomes before and after the intervention between groups that are exposed and unexposed to an intervention. This method can account for secular trends, as well as unobservable differences between the exposed and unexposed groups. This method relies on the assumption that absent the intervention, the groups exposed and unexposed to the intervention would have parallel trends in the outcome. We tested this assumption by visual confirmation and by testing trends in periods pre-ACA for rates of insurance coverage between expansion and non-expansion states.

To perform the difference-in-differences estimation, we used multivariable linear regression models which included an interaction term between a variable for quarter-years before and after Medicaid expansion and a variable for Medicaid expansion status. We performed both an unadjusted analysis. Our unadjusted models included variables indicating pre/post-Medicaid expansion and whether a state expanded Medicaid and an interaction of these variables. The adjusted analyses utilized the interaction term, but also controlled for state and year fixed effects and covariates for age, gender, race/ethnicity, marital status, and employment status. Standard errors were clustered at the state level. We used predictive margins to generate adjusted estimates of insurance coverage and a DiD estimate.

All analyses used sample weights provided by NSDUH to account for the survey's complex sample design and were performed in Stata 15 (Stata Corp). We conducted all our analyses of restricted NSDUH data in a Federal Statistical Restricted Data Center managed by the U.S. Census Bureau. All results were cleared for disclosure by SAMHSA, but the agency did not have a role in study design, analysis, or interpretation of results. Our analysis did not require institutional review board approval because it falls under the Yale University policy for research using de-identified, publicly available data sets. A STROBE checklist for the reporting of cross-sectional studies was completed and is included in supplementary materials.

Expanded discussion of limitations

All outcomes were measured by self-report and cannot be verified through claims or administrative data. They are therefore subject to recall or social desirability bias although self-reported data on criminal justice involvement has been shown to be a valid measure in previous studies. The NSDUH does not survey adults who are institutionalized or unhoused, which may underestimate the proportion of the population with past-year criminal legal involvement. Response rates of the NSDUH as reported above may lead to selection bias, although its use for national estimates, despite this limitation, is widely accepted. DiD methodology cannot account for the possible effect of differences between expansion and non-expansion states that would have coincided temporally with implementation of the ACA and affected our outcome of interest. Despite this limitation DiD methods have been used widely to account for the impact of Medicaid expansion on a variety of outcomes.

© 2022 Howell BA et al. *JAMA Health Forum*.

eTable. State Categorization of Medicaid Expansion by Quarter-Year[1]

State	Medicaid Expansion Status prior to January 2018	Date Implemented	Quarter-year Implemented [3]
Alabama	Not Adopted		
Alaska	Adopted	9/1/2015	Q3 2015
Arizona	Adopted	1/1/2014	Q1 2014
Arkansas	Adopted	1/1/2014	Q1 2014
California	Adopted	1/1/2014	Q1 2014
Colorado	Adopted	1/1/2014	Q1 2014
Connecticut	Adopted	1/1/2014	Q1 2014
Delaware	Adopted	1/1/2014	Q1 2014
District of Columbia	Adopted	1/1/2014	Q1 2014
Florida	Not Adopted		
Georgia	Not Adopted		
Hawaii	Adopted	1/1/2014	Q1 2014
Idaho	Not Adopted[2]		
Illinois	Adopted	1/1/2014	Q1 2014
Indiana	Adopted	2/1/2015	Q1 2015
Iowa	Adopted	1/1/2014	Q1 2014
Kansas	Not Adopted		
Kentucky	Adopted	1/1/2014	Q1 2014
Louisiana	Adopted	7/1/2016	
Maine	Not Adopted[2]		
Maryland	Adopted	1/1/2014	Q1 2014
Massachusetts	Adopted	1/1/2014	Q1 2014
Michigan	Adopted	4/1/2014	Q1 2014
Minnesota	Adopted	1/1/2014	Q1 2014
Mississippi	Not Adopted		
Missouri	Not Adopted[2]		
Montana	Adopted	1/1/2016	Q4 2015
Nebraska	Not Adopted[2]		
Nevada	Adopted	1/1/2014	Q1 2014
New Hampshire	Adopted	8/15/2014	Q3 2014
New Jersey	Adopted	1/1/2014	Q1 2014
New Mexico	Adopted	1/1/2014	Q1 2014
New York	Adopted	1/1/2014	Q1 2014
North Carolina	Not Adopted		
North Dakota	Adopted	1/1/2014	Q1 2014
Ohio	Adopted	1/1/2014	Q1 2014
Oklahoma	Not Adopted[2]		
Oregon	Adopted	1/1/2014	Q1 2014
Pennsylvania	Adopted	1/1/2015	Q4 2014
Rhode Island	Adopted	1/1/2014	Q1 2014
South Carolina	Not Adopted		

© 2022 Howell BA et al. *JAMA Health Forum*.

South Dakota	Not Adopted		
Tennessee	Not Adopted		
Texas	Not Adopted		
Utah	Not Adopted[2]		
Vermont	Adopted	1/1/2014	Q1 2014
Virginia	Not Adopted[2]		
Washington	Adopted	1/1/2014	Q1 2014
West Virginia	Adopted	1/1/2014	Q1 2014
Wisconsin	Not Adopted		
Wyoming	Not Adopted		

1 - https://www.kff.org/health-reform/state-indicator/state-activity-around-expanding-medicaid-under-the-affordable-care-act/

2 – Medicaid expansion not-adopted by 1/1/2018 but subsequently adopted

3 – Used for categorization of quarter-year exposure to Medicaid expansion in DiD statistical analysis

© 2022 Howell BA et al. *JAMA Health Forum*.

STROBE Statement—Checklist of items that should be included in reports of ***cross-sectional studies***

	Item No	Recommendation	Page No
**Title and abstract**	1	(*a*) Indicate the study's design with a commonly used term in the title or the abstract	N/A
		(*b*) Provide in the abstract an informative and balanced summary of what was done and what was found	N/A
**Introduction**			
Background/rationale	2	Explain the scientific background and rationale for the investigation being reported	1
Objectives	3	State specific objectives, including any prespecified hypotheses	1
**Methods**			
Study design	4	Present key elements of study design early in the paper	1
Setting	5	Describe the setting, locations, and relevant dates, including periods of recruitment, exposure, follow-up, and data collection	1, Appendix
Participants	6	(*a*) Give the eligibility criteria, and the sources and methods of selection of participants	1, Appendix
Variables	7	Clearly define all outcomes, exposures, predictors, potential confounders, and effect modifiers. Give diagnostic criteria, if applicable	1
Data sources/ measurement	8*	For each variable of interest, give sources of data and details of methods of assessment (measurement). Describe comparability of assessment methods if there is more than one group	1, Appendix
Bias	9	Describe any efforts to address potential sources of bias	1, Appendix
Study size	10	Explain how the study size was arrived at	1
Quantitative variables	11	Explain how quantitative variables were handled in the analyses. If applicable, describe which groupings were chosen and why	1, Appendix
Statistical methods	12	(*a*) Describe all statistical methods, including those used to control for confounding	1
		(*b*) Describe any methods used to examine subgroups and interactions	N/A
		(*c*) Explain how missing data were addressed	N/A

© 2022 Howell BA et al. *JAMA Health Forum*.

		(*d*) If applicable, describe analytical methods taking account of sampling strategy	Appendix
		(*e*) Describe any sensitivity analyses	N/A
**Results**			
Participants	13*	(a) Report numbers of individuals at each stage of study—eg numbers potentially eligible, examined for eligibility, confirmed eligible, included in the study, completing follow-up, and analysed	N/A
		(b) Give reasons for non-participation at each stage	N/A
		(c) Consider use of a flow diagram	N/A
Descriptive data	14*	(a) Give characteristics of study participants (eg demographic, clinical, social) and information on exposures and potential confounders	2
		(b) Indicate number of participants with missing data for each variable of interest	N/A
Outcome data	15*	Report numbers of outcome events or summary measures	Table 1
Main results	16	(*a*) Give unadjusted estimates and, if applicable, confounder-adjusted estimates and their precision (eg, 95% confidence interval). Make clear which confounders were adjusted for and why they were included	2, Table 1
		(*b*) Report category boundaries when continuous variables were categorized	N/A
		(*c*) If relevant, consider translating estimates of relative risk into absolute risk for a meaningful time period	N/A
Other analyses	17	Report other analyses done—eg analyses of subgroups and interactions, and sensitivity analyses	N/A
**Discussion**			
Key results	18	Summarize key results with reference to study objectives	3
Limitations	19	Discuss limitations of the study, taking into account sources of potential bias or imprecision. Discuss both direction and magnitude of any potential bias	3, Appendix
Interpretation	20	Give a cautious overall interpretation of results considering objectives, limitations, multiplicity of analyses, results from similar studies, and other relevant evidence	3

© 2022 Howell BA et al. *JAMA Health Forum*.

Generalisability	21	Discuss the generalisability (external validity) of the study results	3
**Other information**			
Funding	22	Give the source of funding and the role of the funders for the present study and, if applicable, for the original study on which the present article is based	Acknowledgements

*Give information separately for exposed and unexposed groups.

Note: An Explanation and Elaboration article discusses each checklist item and gives methodological background and published examples of transparent reporting. The STROBE checklist is best used in conjunction with this article (freely available on the Web sites of PLoS Medicine at http://www.plosmedicine.org/, Annals of Internal Medicine at http://www.annals.org/, and Epidemiology at http://www.epidem.com/). Information on the STROBE Initiative is available at www.strobe-statement.org.

© 2022 Howell BA et al. *JAMA Health Forum*.
